# Refraction and How to Refract

**Published:** 1900-01

**Authors:** 


### *BOOK REVIEWS, PAMPHLETS, EXCHANGES.*

---

### *BOOK REVIEWS.*

**[Contributions solicited for review.]**

---

REFRACTION AND HOW TO REFRACT. By James Thorington, A.M., M.D., Philadelphia, Adjunct Professor of Ophthalmology in the Philadelphia Polyclinic; Assistant Surgeon to Will's Eye Hospital; Associate Member of American Ophthalmological Society, etc.

This work of 301 pages is from the pen of one well-known for his work in Refraction, and especially that portion of it dealing with Retinoscopy. As would naturally be expected from one who has given this subject so much thought and study, we have presented to us an exceedingly instructive and clear exposition of this subject.

There are several good works on the subject of Refraction, most of which are too voluminous and scientific to be of much practical value to the men just starting in to study the eye, but in this book Dr. Thorington has given an exceedingly clear exposition, terse and concise, and in language which ought to be understood by every student. Its author is well-known also for his previous little brochure on Retinoscopy, a test which, when combined with test lenses, makes the refractive examination almost as perfect as can be. The man who does not use retinoscopy is not a competent refractionist, for with small children, the illiterate and demented it is our one sole authority, and after it has once been mastered how quickly we can detect the amount of refractive error.

The last chapter in the book is devoted to the practical subject of the "proper fitting of spectacles and eye-glasses." This is one of the most valuable chapters in the book, because it educates the ophthalmologist to know when the optician has made a mistake, or at least, forces him to be more correct in his fittings. The contents of the book are most excellently arranged. R.

